# Supplementary material for: Routes of Zika virus dissemination in the testis and epididymis of immunodeficient mice
Source: Nat Commun. 2018 Dec 17;9:5350. doi: 10.1038/s41467-018-07782-x (PMC6297220; doi:10.1038/s41467-018-07782-x)
Supplement: Supplementary file 1 — Supplementary Information [file 41467_2018_7782_MOESM1_ESM.docx]

**Supplementary Information**

**Routes of Zika virus dissemination in the testis and epididymis of immunodeficient mice**

**Tsetsarkin et al**

**Supplementary Figures**

|  |
| --- |
| **Supplementary Figure 1. miRNA targeting of 3’NCR of ZIKV.**  **a** - Schematic representation of miRNA targets inserted into ZIKV genome (white boxes). **b -** annotated sequences of the 5’ terminus of the 3’NCR for viruses used in the study. The sequence of ZIKV-NS3m is on the top. Arrows and underlined sequences highlight the positions of miRNA target insertions in the 3’NCR. NsiI, XhoI and ClaI - restriction endonuclease sites that were used for miRNA target insertion and for construction of the depicted infectious clones. |

|  |
| --- |
| **Supplementary Figure 2. Growth kinetics of 2×scr and 2×202(T) in the testis of AG129 male mice.**  Adult AG129 mice were infected ip with 10^6^ pfu of 2×scr or 2×202(T) and were sacrificed at various intervals of post infection. Mean viral titer ± standard deviation (SD, shown as error bars) in the testicular pair homogenates (n = 3-7 per time point) was determined by titration in Vero cells. Differences between the growth kinetics of 2×scr and 2×202(T) viruses was compared using two-way ANOVA to generate the reported p-value.  **Note 1:** the data, which was used to create growth kinetics of 2×scr and 2×202(T) in the testis, was also used to generate Fig. 1f, 2f, 2g**.**  **Note 2:** due to miRNA target instability of 2×202(T) in the testis, the 3’NCR of virus in every testicular sample was sequenced. Only testicular samples with intact virus sequence were used to evaluate growth kinetics of 2×202(T), hence the name Te-2×202(T)-stb. |

| 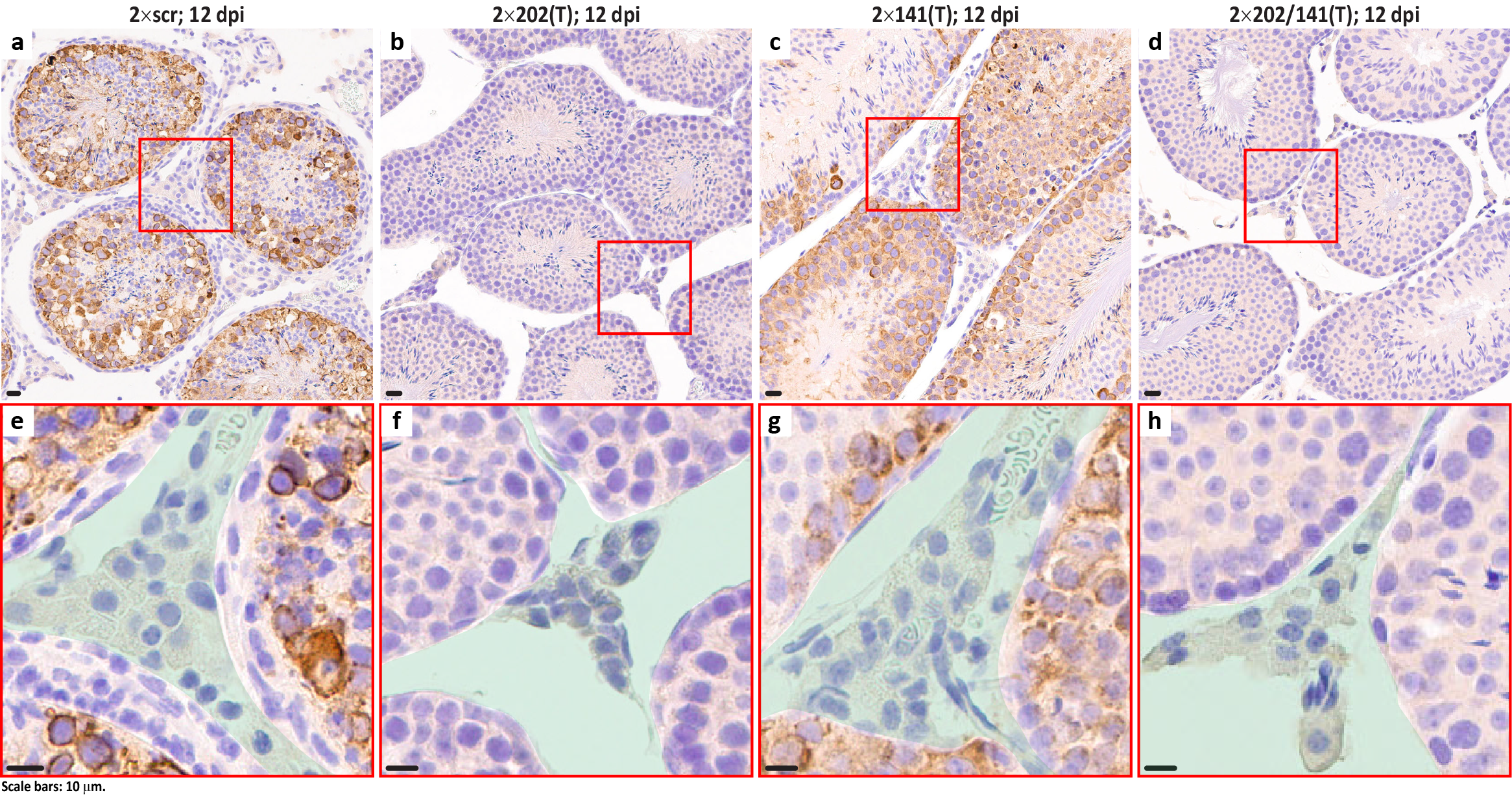 |
| --- |
| **Supplementary Figure 3. Clearance of miRNA-targeted viruses from the testicular interstitium at 12 dpi**. (**a**-**d**) Immunostaining for ZIKV antigen in the testes of mice infected ip with indicated viruses. Respective boxed areas are enlarged in **e** – **h** and interstitial compartments are highlighted by green overlay. Note (i) a complete absence of the ZIKV antigen within the testicular interstitium at this time point regardless of the inoculated virus; and (ii) a pancellular damage induced by 2×scr virus within the seminiferous tubules (**a**). Scale bars: 10 µm. |

| 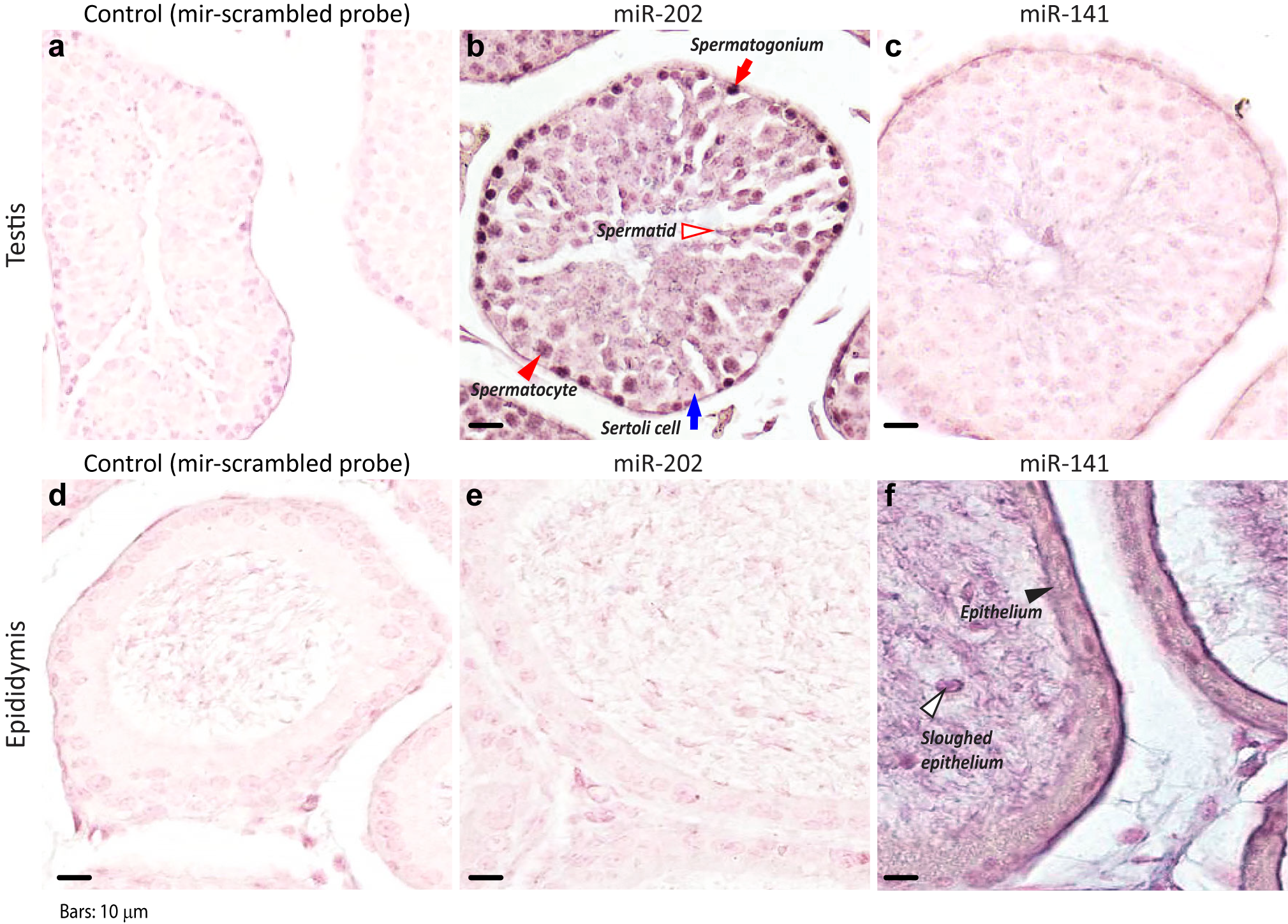 |
| --- |
| **Supplementary Figure 4. Detection of mir-202-5p and mir-141-3p in the testis and epididymis of AG129 mice by in situ hybridization.**  The negative control (mir-scrambled probe) panels (**a** and **d**) on the left for both, testis (**a**) and epididymis (**d**), are shown for comparison of signal over background. Counterstain is nuclear fast red (pink).  Note (i) a positive mir-202-5p signal (blue) in the indicated cells of the seminiferous tubules (**b**); and (ii) a positive mir-141-3p signal (blue) in the epididymal epithelium and a putative sloughed epithelium within the lumen of epididymal duct (**e**). The signals for mir-202-5p in the epididymis and for mir-141-3p in the testis are not above the background (**c** and **f**). Scale bars: 10 µm. |

|  |
| --- |
| **Supplementary Figure 5. A single (out of 6 tested) testicle infected with 2×202(T) at 12 dpi that had detectable ZIKV antigen in the seminiferous tubules.**  Adult AG129 mice were infected ip with 10^6^ pfu of 2×202(T) virus and sacrificed at 12 dpi as described in Fig. 3. Images showing distribution of 2×202(T) in the testis at low (1.25x) and high (20x) magnification [n=1 out of 6 testicles from 3 mice) at 12 dpi.  Scale bar equals 1mm at 1.25x and 50 µm at 20x magnifications. |

| 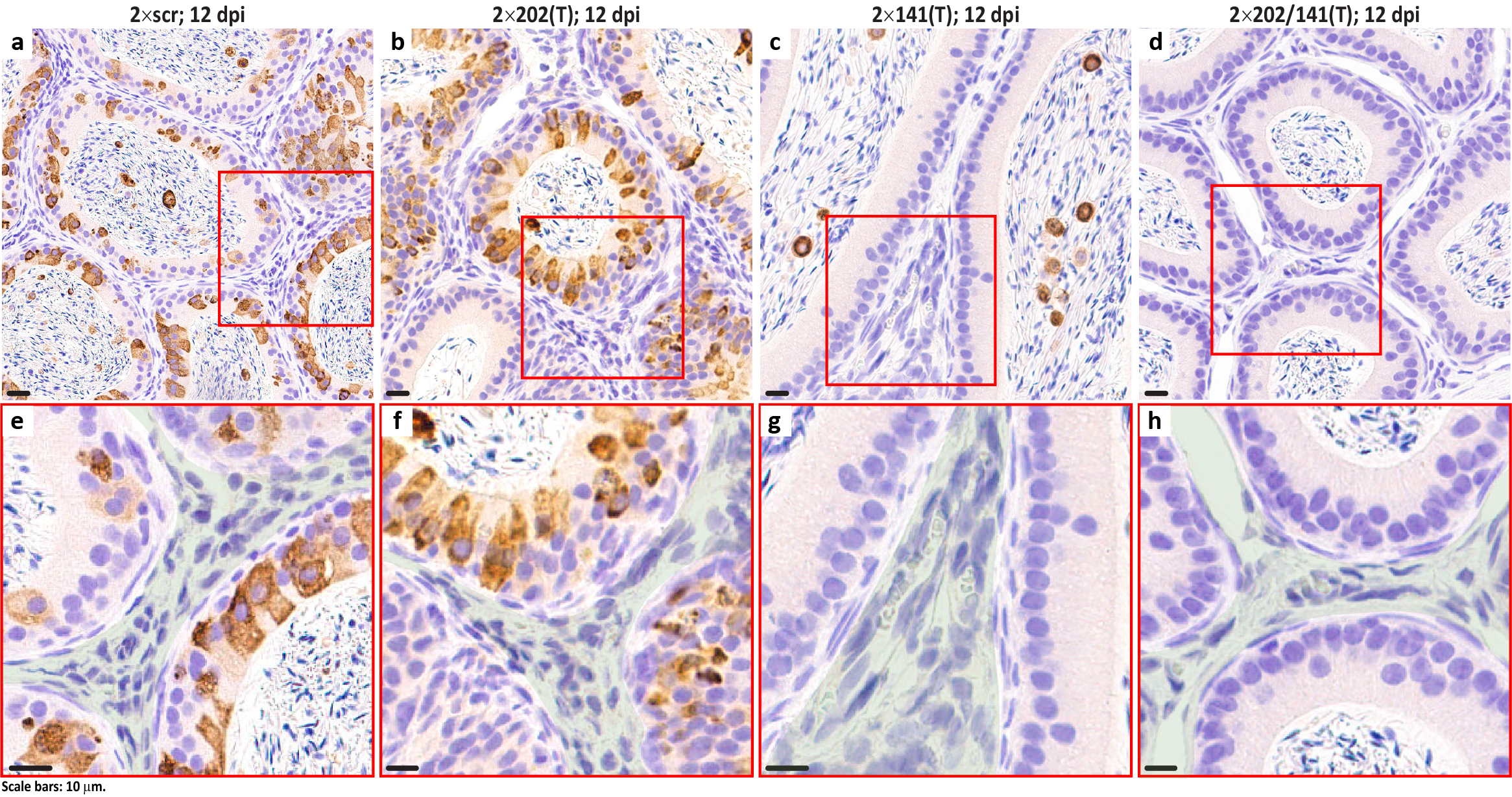 |
| --- |
| **Supplementary Figure 6. Clearance of miRNA-targeted viruses from epididymal interstitium at 12 dpi**. (**a**-**d**) Immunostaining of ZIKV antigen in the epididymides of mice infected ip with indicated viruses. Respective boxed areas are enlarged in **e**–**h** and interstitial compartments are highlighted by green overlay. Note (i) a complete absence of the ZIKV antigen within the epididymal interstitium at this time point regardless of the inoculated virus; and (ii) a damage to the epididymal epithelium induced by ZIKV replication that manifest in increased sloughing of cells into the ductal lumen (**a**; top right corner). Scale bars: 10 µm. |

|  |
| --- |
| **Supplementary Figure 7. Sequencing electropherograms of 2×202(T) virus isolated from the testis, epididymis, and brain of a mouse #12 at 12 dpi.**  Adult AG129 mice were infected ip with 10^6^ pfu of 2×202(T) viruses and were sacrificed at 12 dpi.  **On the top** - annotated sequence of the 5’ terminus of the 3’NCR for 2×202(T) virus showing two mir-202-5p targets. Nucleotide sequence of the 2×202(T) genome, which were deleted in the testicular samples of a mouse #12 (see Supplementary Table 1), are underlined. Red arrow highlights the 3’-end of the deleted sequence.  **On the bottom** –sequencing electropherograms of 2×202(T) cDNA genome generated using RNA isolated from the testis, epididymis, and brain of mouse #12 (see Supplementary Table 1). Red arrows highlight the 3’-end of deleted sequence identified in 2×202(T) isolated from the testicular sample. |

|  |
| --- |
| **Supplementary Figure 8. Sequencing electropherograms of 2×141(T) virus isolated from the testis, epididymis, and brain of mouse #5 at 12 dpi.**  Adult AG129 mice were infected ip with 10^6^ pfu of 2×141(T) viruses and sacrificed at 12 dpi.  **On the top** - annotated sequence of the 5’ terminus of the 3’NCR for 2×141(T) virus showing both mir-141-3p targets. Nucleotide sequence of the 2×141(T) virus that were deleted in ~40% of virus genomes (mixed population) in the epididymis sample from a mouse #2 (see Supplementary Table 2) are underlined. Red arrow highlights the 3’-end of the genome heterogeneity.  **On the bottom** –sequencing electropherograms of 2×141(T) cDNA genome generated using RNAs isolated from the testis, epididymis, and brain of mouse #2 (see Supplementary Table 2). Red arrows highlight the 3’-end of sequence affected by 2×141(T) virus genome heterogeneity, which was identified in the epididymis sample from mouse #5. |

|  |
| --- |
| **Supplementary Figure 9. Synergistic cooperation between mir-202-5p and mir-141-3p targets, but not the insertion of long heterologous sequence into 3’NCR of ZIKV, is responsible for 2×202/141(T) virus attenuation in the testis and epididymis of AG129 mice.**  **(a)** Annotated sequences of the 2×202/141(T) and scr(Long) viruses.  **(b-d)** AG129 mice were infected ip with 10^6^ pfu of 2×202/141(T) or scr(Long) virus and sacrificed at 12 dpi. Mean viral titer ± SD (shown as error bars) in the testis (**b**), epididymis (**c**), and brain (**d**) was determined by titration in Vero cells. The dashed lines indicate the limit of virus detection: 1.7 log_10_(pfu/g) of brain or testis, and 0.7 log_10_(pfu/mouse) for epididymis. Differences between the titer of scr(Long) and 2×202/141(T) viruses in mouse organs were compared using one-way ANOVA (*** p<0.001, * p<0.05).  **Note 1:** mice infected with 2×202/141(T) virus were divided into 2 groups (stb and mut) based on genetic stability of miRNA target sequences in the testis-isolated viruses at 12 dpi. For both groups of mice (stb and mut), the titers of 2×202/141(T) viruses in the testis, epididymis, and brain are presented separately.  **Note 2:** the data for 2×202/141(T) virus presented in this figure was also used to generate Fig. 2g, 2h, 4c. |

|  |
| --- |
| **Supplementary Figure 10. Validation of the dual route dissemination mechanism for ZIKV in the epididymis using targets for various testis- and epididymis-specific miRNAs.**  (**a-b**) relative expression of mir-465a-3p and mir-200a-3p in the testis (**a**) and epididymis (**b**) of AG129 mice. (**c**) Annotated sequences of the 5’ terminus of the 3’NCR for viruses used in the study. (**c**) List of viruses with inserted miRNA targets. Virus titers in Vero cell supernatant at 3 days post plasmid DNA transfection is expressed as log_10_(pfu/mL). (**e-k)** Adult AG129 mice were infected ip with 10^6^ pfu of 2×scr or miRNA-targeted ZIKV and were sacrificed at 3 and 12 dpi. Mean viral titer ± SD (shown as error bars) in the serum at 1 dpi (**e**), spleen at 3 dpi (**f**), testis at 3 dpi (**g**), epididymis at 3 dpi (**h**), brain at 12 dpi (**i**), testis at 12 dpi (**j**) and epididymis at 12 dpi (**k**) was determined by titration in Vero cells. Mice infected with 2×465a(T), and 2×202/202a(T) viruses were divided into 2 groups based on miRNA target sequence stability in the virus genome isolated from the testis of these mice at 12 dpi. For both groups of mice (stb and mut) the titers of these viruses in the brain and testis are presented separately. All viruses recovered at 12 dpi from the testis of mice (n=4) infected with 2×465a/141(T) were stable. Therefore, the data for only the group of Te-2×465a/141(T)-stb (and not for the group Te-2×465a/141(T)-mut) is presented in panels (**i-k**). The dashed lines indicate the limit of virus detection: 1.5 log_10_(pfu/mL) of serum (**e**), 1.7 log_10_(pfu/g) of spleen, brain or testis (**f, g, j, j**) and 0.7 log_10_(pfu/mouse) for epididymis (**h, k**). Differences between the titer of 2×scr and the titer of each of miRNA-targeted viruses in mouse serum or organs were compared using one-way ANOVA (*** p<0.001).  **Experiment description:** |

It is possible that attenuation of the 2×202/141(T) virus in the epididymis was not miRNA-mediated, but it was attributed to unknown phenomena associated with a particular sequence inserted into the 3’NCR of the 2×202/141(T). This would invalidate the ‘dual route dissemination’ hypothesis proposed to explain ZIKV invasion into epididymis. However, if the miRNA-mediated mechanism of epididymal attenuation of the 2×202/141(T) is valid, then any ZIKV clones targeted for combination of any testis- and epididymis-specific miRNAs should also be simultaneously attenuated in the testis and in epididymis, as long as selected miRNAs independently restrict ZIKV replication in the testis and in the epididymal epithelium. Stated in another way, attenuation of the 2×202/141(T) in the epididymis was not a unique event, but was a common consequence of simultaneous restrictions of both hematogenous/lymphogenous and excurrent testicular routes of infection by dual miRNA targeting.

To confirm this, first we generated two additional ZIKV clones [2×465a(T) and 2×200c(T)] which contain targets for miRNAs selectively expressed in the testis (mir-465a-3p) or in the epididymis (mir-200c-3p) (Supplementary Fig. 10a, 10b). The mir-465a-3p is specifically expressed in the testis^1,2^. It is a mouse-specific miRNA, which cannot be used in human vaccine research applications, but it is a useful tool for proof-of-principal studies. The mir-200c-3p is expressed in both humans and in mice. It is a member of mir-200 family^1^, which also include mir-141-3p, and it is expressed with the later from a common promoter^3,4^ . Similar to mir-141-3p, the mir-200c-3p also regulates maintenance of epithelial cell phenotype^3,5^, implying that mir-200c-3p is primarily expressed in the epididymal epithelium.

Similar to 2×202(T) and 2×141(T) viruses, the replication of 2×465a(T) and 2×200c(T) was indistinguishable from 2×scr during the early course of viral infection in the serum, spleen, testis and epididymis of AG129 mice (Supplementary Fig. 10e-10h). Moreover, at 12 dpi the titer of both viruses was also similar to 2×scr in the brain and epididymis (Supplementary Fig. 10i, 10k), and both viruses isolated from these organs remained stable. The titer of 2×465a(T) virus in the testis of Te-2×465a(T)-stb mice, but not the Te-2×465a(T)-mut mice, was significantly reduced compared to the 2×scr (Supplementary Fig. 10j, also see Supplementary Table 4). In contrast, replication of the 2×200c(T) in the testis at 12 dpi was not attenuated and the virus remained stable (Supplementary Fig. 10j).

Next, we modified the 2×202/141(T) virus by replacing targets for testis specific mir-202-5p with targets for mir-465a-3p, generating 2×465a/141(T) virus. Reciprocally, targets for epididymis specific mir-141-3p in virus 2×202/141(T) were replaced with two targets for mir-200c-3p, generating 2×202/200c(T) (Supplementary Fig. 10c). Both 2×465a/141(T) and 2×202/200c(T) replicated indistinguishably from 2×scr in the serum and mouse organs during early infection (Supplementary Fig. 10e-10h) and in the brain at 12 dpi (Supplementary Fig. 10i). However, in contrast to 2×465a(T) and 2×200c(T) at the 12 dpi, accumulation of both 2×465a/141(T) and 2×202/200c(T) in the epididymis was significantly attenuated only in mice that preserved stable miRNA targets sequences in their testis (Supplementary Fig. 10k).

Escape mutants for the 2×465a/141(T) virus isolated form the mouse testis at 12 dpi (n=4) we not detected, but mutations were found in the 3 out 8 of testis samples derived from the 2×202/200c(T)-infected mice. The titer of 2×202/200c(T) virus in the testes and epididymides of Te-2×202/200c(T)-mut mice were similar to the titer of 2×scr virus in these organs (Supplementary Fig. 10j). Sequence analysis showed that in the majority (2/3) of virus samples, the size and location of the deletions were identical in testis- and epididymis-derived viruses isolated from the same animal (see Supplementary Table 5; mouse #1 and #4). These deletions always affected sequences for both mir-202-5p targets. However, in one of the two mice the deletion preserved intact one 3’-terminal mir-200c-3p target (see Supplementary Table 5; mouse #1).

|  |
| --- |
| **Supplementary Figure 11. C/3’NCR-mir(T) virus.**  **(a)** Schematic representation of C/3’NCR-mir(T) virus genome: dCGR – duplicated capsid gene region; C-trn(50AA) – truncated C gene; * - open reading frame (ORF) shifting mutation (Fr Sh) in the C-trn; +1 and -1 are insertion and deletion of one nucleotide, respectively; C-opt is a full-length cDNA copy of C gene containing synonymous mutations introduced in each AA codon (except ATG and TGG); colored boxes indicate miRNA targets for mir-9-5p (cherry), mir-141-3p (purple), mir-202-5p (blue), mir-124-3p (red); 2A - autoprotease 2A from FMDV; the curved arrow indicates position of 2A protease cleavage. (**b** and **c)** The annotated sequences of dCGR (**b**) and 5’ terminus of the 3’NCR (**c**) of C/3’NCR-mir(T) virus. XhoI and ClaI - restriction endonuclease sites that were used for miRNA target insertion and for construction of the C/3’NCR-mir(T). |

|  |
| --- |
| **Supplementary Figure 12. C/3’NCR-scr virus.**  **(a)** Schematic representation of C/3’NCR-scr virus genome. Designations for C-trn(50AA), C-opt, *, curved arrow and 2A box are the same as in Figure S11; striped cherry and red boxes indicate mutated targets for mir-9-5p and mir-124-3p, respectively; striped green box indicates scr sequence. Gray box indicates random sequence of 21 nt. (**b** and **c)** The annotated sequences of dCGR (**b**) and 5’ terminus of the 3’NCR (**c**) of C/3’NCR-scr virus. Capital letters indicate synonymous mutation in mir-9-5p and mir-124-3p targets. XhoI and ClaI - restriction endonuclease sites that were used for construction of C/3’NCR-scr. |

|  |
| --- |

**Supplementary Figure 13. Protective efficacy of C/3’NCR-mir(T) and C/3’NCR- scr viruses against challenge with wt ZIKV in adult AG129 mice.**

**(a)** Experimental design of immunogenicity study. Adult AG129 mice were mock-inoculated or virus-infected ip with 10^5^ pfu of C/3’NCR-mir(T) or C/3’NCR-scr. At 29 dpi, mock- or virus-inoculated mice were challenged with 10^5^ pfu of wt ZIKV (Paraiba_01/2015) and monitored for an onset of neurological disease (paralysis) for 27 dpc. Mice were bled at 28 and 56 dpi to determine ZIKV specific NA titer in the serum (see Fig. 5j, 5i for the data) and at 2 dpc (31 dpi) to evaluate viremia. (**b)** Survival of immunized mice after challenge with wt ZIKV. (**c)** The mean titer ± SD (shown as error bars) of wt ZIKV in the serum of mice at 2 days post challenge. The dashed lines indicate the limit of virus detection [1.5 log_10_(pfu/mL)].

| \|  \| \| --- \| \| **Supplementary Figure 14. Relative expression of the selected miRNAs in the whole testis (a) and in the type A spermatogonia (b), pachytene spermatocytes (c), and round spermatids (d).**  Relative expressions were calculated as a ratio of the normalized number of reads for each miRNA to the number of reads for the miRNA, which was least expressed among the tested miRNAs in the given organ/cells type. The original data for miRNA expression profile in the mouse testis/ testis-derived cells was obtained in reference^2^. \| |
| --- | --- | --- |

**Supplementary Tables**

**Supplementary Table 1.** Virus titers and stability of 2×202(T) in the testis, epididymis, and brain of AG129 mice at 12 dpi.

| **Mouse**  **ID** | **Testis** | | | **Epididymis** | | **Brain** | |
| --- | --- | --- | --- | --- | --- | --- | --- |
|  | **Titer ^a^** | **Stability** | **Deletion ^c^** | **Titer ^b^** | **Stability** | **Titer ^a^** | **Stability** |
| #1 | 2.48 | stb | - | NT | NT | 5.98 | stb |
| #2 | 7.59 | mut | 1-135 nt | NT | NT | 6.18 | stb |
| #3 | 4.47 | stb | - | 6.29 | stb | 7.33 | stb |
| #4 | 3.39 | stb | - | 5.48 | stb | 7.87 | stb |
| #5 | 6.97 | mut | 27-52 nt | 4.91 | stb | 6.88 | stb |
| #6 | 3.43 | stb | - | 5.12 | stb | 7.87 | stb |
| #7 | 3.11 | stb | - | 6.26 | stb | 6.57 | stb |
| #8 | 4.26 | stb | - | 6.51 | stb | 7.18 | stb |
| #9 | 5.90 | mut | 19-69 nt | 6.47 | stb | 7.93 | stb |
| #10 | 5.68 | mut | 18-136 nt | 5.51 | stb | 7.98 | stb |
| #11 | 6.41 | mut | 21-66 nt | 5.56 | stb | 7.76 | stb |
| #12 | 7.07 | mut | 21-61 nt | 5.83 | stb* | 7.38 | stb |

**a** – Virus titer is expressed as log_10_(pfu/g of tissue)

**b** – Virus titer is expressed as log_10_(pfu/mouse)

**c** – Positions of the deleted nucleotides in the 3’NCR of 2×202(T)

NT- not tested

stb – stable (no mutations were found in the miRNA targeted region)

mut – escape mutant

* partial instability; see Supplementary Fig. 7 for details

**Supplementary Table 2.** Virus titers and stability of 2×141(T) in the testis, epididymis, and brain of AG129 mice at 12 dpi.

| **Mouse**  **ID** | **Testis** | | **Epididymis** | | **Brain** | |
| --- | --- | --- | --- | --- | --- | --- |
|  | **Titer ^a^** | **Stability** | **Titer ^b^** | **Stability** | **Titer ^a^** | **Stability** |
| #1 | 7.30 | stb | NT | NT | 8.28 | stb |
| #2 | 7.85 | stb | NT | NT | 7.30 | stb |
| #3 | 7.30 | stb | NT | NT | 6.70 | stb |
| #4 | 7.43 | stb | 4.48 | stb | 7.08 | stb |
| #5 | 7.36 | stb | 5.55 | stb/mut* | 6.14 | stb |
| #6 | 7.42 | stb | 6.28 | stb | 6.52 | stb |
| #7 | 8.00 | stb | 5.09 | stb | 7.06 | stb |
| #8 | 7.90 | stb | 5.53 | stb | 6.38 | stb |

**a** – Virus titer is expressed as log_10_(pfu/g of tissue)

**b** – Virus titer is expressed as log_10_(pfu/mouse)

NT - not tested

stb – stable (no mutations were found in the miRNA targeted region)

mut – escape mutant

* partial instability; see Supplementary Fig. 8 for details

**Supplementary Table 3.** Infectious titers and stability of 2×202/141(T) in the testis, epididymis, and brain of AG129 mice at 12 dpi.

| **Mouse**  **ID** | **Testis** | | | **Epididymis** | | | **Brain** | |
| --- | --- | --- | --- | --- | --- | --- | --- | --- |
|  | **Titer ^a^** | **Stability** | **Deletion ^c^** | **Titer ^b^** | **Stability** | **Deletion ^c^** | **Titer ^a^** | **Stability** |
| #1 | 4.61 | stb | - | 1.60 | NT | - | 4.18 | stb |
| #2 | 6.36 | mut | 20-95* | 5.03 | mut | 20-95* | 6.98 | stb |
| #3 | 6.11 | mut | 24-65** | 4.08 | mut | 24-65** | 5.47 | stb |
| #4 | 7.74 | mut | 13-86*** | 5.26 | mut | 13-86*** | 6.51 | stb |
| #5 | 3.14 | stb | - | 2.11 | NT | - | 8.02 | stb |
| #6 | 3.37 | stb | - | 2.37 | NT | - | 8.39 | stb |
| #7 | 2.46 | stb | - | 1.81 | NT | - | 5.51 | stb |
| #8 | 4.00 | stb | - | 2.36 | NT | - | 7.48 | stb |
| #9 | 6.02 | mut | 1-176**** | 4.05 | mut | 1-176**** | 7.74 | stb |

**a** – titer is expressed as Log_10_(pfu/g)

**b** – titer is expressed as Log_10_(pfu/mouse)

**c** – positions of the deleted nucleotides in the 3’NCR of 2×202/141(T) are indicated

stb – stable (no mutations were found in the miRNA targeted region)

mut – escape mutant

NT - not tested

* - Deletion in the 2×202/141(T) virus isolated from the testis and epididymis of **mouse #2**

mir-202-5p(T) mir-141-3p(T) XhoI mir-202-5p(T) ClaI

TAAGCACCAA**caaagaagtata~~tgcataggaaccatctttaccagacagtgtta~~*~~CTCGAG~~*~~caaagaagtatatgcataggaa~~*~~ATCGAT~~***~~t~~

mir-141-3p(T)

**~~ccatcttta~~ccagacagtgttaa**TGTTGTC

** - Deletion in the 2×202/141(T) virus isolated from the testis and epididymis of **mouse #3**

mir-202-5p(T) mir-141-3p(T) XhoI mir-202-5p(T) ClaI

TAAGCACCAA**caaagaagtatatgca~~taggaaccatctttaccagacagtgtta~~*~~CTCGAG~~*~~caaagaag~~tatatgcataggaa*ATCGAT***t

mir-141-3p(T)

**ccatctttaccagacagtgttaa**TGTTGTC

***- Deletion in the 2×202/141(T) virus isolated from the testis and epididymis of **mouse #4**

mir-202-5p(T) mir-141-3p(T) XhoI mir-202-5p(T) ClaI

TAAGCACCAA**caaag~~aagtatatgcataggaaccatctttaccagacagtgtta~~*~~CTCGAG~~*~~caaagaagtatatgcataggaa~~*~~ATCGAT~~***~~t~~

mir-141-3p(T)

**ccatctttaccagacagtgttaa**TGTTGTC

****- Deletion (5’-end) in the 2×202/141(T) virus isolated from the testis and epididymis of **mouse #9**

mir-202-5p(T) mir-141-3p(T) XhoI mir-202-5p(T) ClaI

TAA~~GCACCAA~~**~~caaagaagtatatgcataggaaccatctttaccagacagtgtta~~*~~CTCGAG~~*~~caaagaagtatatgcataggaa~~*~~ATCGAT~~***~~t~~

mir-141-3p(T)

**~~ccatctttaccagacagtgttaa~~**~~TGTTGTC~~

**Supplementary Table 4.** Infectious titers and stability of 2×465a(T) in the testis, epididymis, and brain of AG129 mice at 12 dpi.

| **Mouse**  **ID** | **Testis** | | | **Epididymis** | | **Brain** | |
| --- | --- | --- | --- | --- | --- | --- | --- |
|  | **Titer ^a^** | **Stability** | **Deletion ^c^** | **Titer ^b^** | **Stability** | **Titer ^a^** | **Stability** |
| #1 | 7.44 | mut | 11-69 nt * | 5.27 | stb | 7.45 | stb |
| #2 | 3.74 | stb | - | 5.98 | stb | 6.06 | stb |
| #3 | 3.97 | stb | - | 6.24 | stb | 7.11 | stb |
| #4 | 4.15 | stb | - | 6.43 | stb | 7.54 | stb |
| #5 | 5.62 | stb | - | 6.71 | stb | 6.09 | stb |
| #6 | 4.87 | stb | - | 5.60 | stb | 7.95 | stb |
| #7 | 3.04 | stb | - | 6.28 | stb | 7.27 | stb |

**a** – titer is expressed as Log_10_(pfu/g)

**b** – titer is expressed as Log_10_(pfu/mouse)

**c** – positions of the deleted nucleotides in the 3’NCR of 2×465a(T)

NT - not tested

stb – stable (no mutations were found in the miRNA targeted region)

mut – escape mutant

* - Deletion in the virus isolated from the testis of **mouse #1**

mir-465a-3p(T) NsiI mir-465a-3p(T)

TAAGCACCAA**Tct~~acttagaaaggccctgatc~~*~~ATGCAT~~***~~ACCAATCTTAA~~**~~Tctacttagaaaggccctgatc~~**~~T~~GTTGTC

**Supplementary Table 5.** Infectious titers and stability of 2×202/200c(T) in the testis, epididymis, and brain of AG129 mice at 12 dpi.

| **Mouse**  **ID** | **Testis** | | | **Epididymis** | | | **Brain** | |
| --- | --- | --- | --- | --- | --- | --- | --- | --- |
|  | **Titer ^a^** | **Stability** | **Deletion ^c^** | **Titer ^b^** | **Stability** | **Deletion ^c^** | **Titer ^a^** | **Stability** |
| #1 | 7.317 | mut | 18-79* | 6.08 | mut | 18-79* | 18-79 nt* | stb |
| #2 | 4.08 | stb | - | 2.35 | NT | - | 6.52 | stb |
| #3 | 2.93 | stb | - | 1.77 | NT | - | 5.58 | stb |
| #4 | 5.62 | mut | 18-175** | 4.37 | mut | 18-175** | 6.06 | stb |
| #5 | 7.45 | mut | 34-93*** | 6.09 | mut | 18-193*** | 6.45 | stb |
| #6 | 4.57 | stb | - | 3.28 | NT | - | 7.26 | stb |
| #7 | 2.00 | stb | - | 1.81 | NT | - | 6.36 | stb |
| #8 | 3.50 | stb | - | 2.54 | NT | - | 6.68 | stb |

**a** – titer is expressed as Log_10_(pfu/g)

**b** – titer is expressed as Log_10_(pfu/mouse)

**c** – positions of the deleted nucleotides in the 3’NCR of 2×202/200c(T) are indicated

stb – stable (no mutations were found in the miRNA targeted region)

mut – escape mutant

NT - not tested

* - Deletion in the 2×202/200c(T) virus isolated from the testis and epididymis of **mouse #1**

mir-202-5p(T) mir-200c-3p(T) XhoI mir-202-5p(T) ClaI

TAAGCACCAAT**caaagaagt~~atatgcataggaatccatcattacccggcagtatta~~*~~CTCGAG~~*~~caaagaagtatatgcatagg~~aa*ATCGA***

mir-200c-3p(T)

**tccatcattacccggcagtattA**TGTTGTC

** - Deletion (5’-end) in the 2×202/200c(T) virus isolated from the testis and epididymis of **mouse #4**

mir-202-5p(T) mir-200c-3p(T) XhoI mir-202-5p(T) ClaI

TAAGCACCAAT**caaagaagt~~atatgcataggaatccatcattacccggcagtatta~~*~~CTCGAG~~*~~caaagaagtatatgcataggaa~~*~~ATCGA~~***

mir-200c-3p(T)

**~~tccatcattacccggcagtattA~~**~~TGTTGTC~~

*** - Point mutation (red) and a deletion in the 2×202/200c(T) virus isolated from the testis of **mouse #5**

mir-202-5p(T) mir-200c-3p(T) XhoI mir-202-5p(T) ClaI

TAAGCACCAAT**caaagaagtat(a**🡪**g)tgcataggaatcc~~atcattacccggcagtatta~~*~~CTCGAG~~*~~caaagaagtatatgcataggaa~~*~~A~~***

ClaI mir-200c-3p(T)

***~~TCGA~~*~~tccatca~~ttacccggcagtattA**TGTTGTC

**** - Deletion (5’-end) in the 2×202/200c(T) virus isolated from the epididymis of **mouse #5**

mir-202-5p(T) mir-200c-3p(T) XhoI mir-202-5p(T) ClaI

TAAGCACCAAT**caaagaagt~~atatgcataggaatccatcattacccggcagtatta~~*~~CTCGAG~~*~~caaagaagtatatgcataggaa~~*~~ATCGA~~***

mir-200c-3p(T)

**~~tccatcattacccggcagtattA~~**~~TGTTGTC~~

**Supplementary Table 6.** List of primers used in the study

| **Name ^a^** | **Sequence ^b^** |
| --- | --- |
| ZV-1-F | AGTTGTTGATCTGTGTGAATC |
| ZV-451-R | AGGCCAACAATTCCGACACT |
| ZV-10044-F | GGGAGAACTACCTGGTCAATC |
| ZV-10722-R | GGTCTTTCCCAGCGTCAATATG |

**a** – numbers in the primer name correspond to a position of the 5’-terminal nucleotide of the primer in the genome of ZIKV (strain Paraiba_01/2015). F - denotes forward and R – denotes reverse orientation of the primer with regard to the positive sense genome of ZIKV.

**b** – Sequences of the primers are given in the 5’🡪3’ orientation.

**Supplementary References**

1. <http://mirbase.org/>. Vol. 2018 (2018).

2. Chen, J. *et al.* MicroRNA-202 maintains spermatogonial stem cells by inhibiting cell cycle regulators and RNA binding proteins. *Nucleic Acids Res* **45**, 4142-4157 (2017).

3. Burk, U. *et al.* A reciprocal repression between ZEB1 and members of the miR-200 family promotes EMT and invasion in cancer cells. *EMBO Rep* **9**, 582-9 (2008).

4. Batista, L., Bourachot, B., Mateescu, B., Reyal, F. & Mechta-Grigoriou, F. Regulation of miR-200c/141 expression by intergenic DNA-looping and transcriptional read-through. *Nat Commun* **7**, 8959 (2016).

5. Park, S.M., Gaur, A.B., Lengyel, E. & Peter, M.E. The miR-200 family determines the epithelial phenotype of cancer cells by targeting the E-cadherin repressors ZEB1 and ZEB2. *Genes Dev* **22**, 894-907 (2008).
